# Metagenomic analysis of bacterial and viral communities of *Aedes aegypti* and *Aedes albopictus*

**DOI:** 10.1016/j.jgeb.2025.100643

**Published:** 2026-01-03

**Authors:** Shahzadi Asia Nadeem, Ijaz Ali, Hazrat Hussain, Ihsan Ullah, Wajid Ali, Khalid J. Alzahrani, Hamid Ali, Zarak Imtiaz Khan, Kasim Sakran Abass, Rafi ur Rahman

**Affiliations:** a Department of Biosciences, COMSATS University Islamabad, Pakistan; b Center for Applied Mathematics & Bioinformatics (CAMB), Gulf University for Science & Technology, Hawally, Kuwait; c Department of Biotechnology, University of Swabi, Swabi. Pakistan; d Ibadat International University, Islamabad. Pakistan; e Department of Clinical Laboratories Sciences, College of Applied Medical Sciences, Taif University, Saudi Arabia; f Department of Physiology, Biochemistry, and Pharmacology, University of Kirkuk, Iraq; g Laboratory of Biochemistry and Physiology of Insects, Oswaldo Cruz Foundation. Rio de Janeiro, Brazil

**Keywords:** *Aedes aegypti*, Microbiota, *Human endogenous retrovirus K*, Vector biology, Vector competence

## Abstract

**Background:**

The complicated relationship between the *Aedes* mosquito microbiome, arbovirus transmission and essential physiological processes, is extremely important. Microbial community plays a vital role in shaping vector biology, impacting critical aspects such as parasite replication within the vector, vector longevity, and ultimately, vector competence. Understanding the composition and function of the *Aedes* microbiome is therefore crucial for developing novel strategies to control arboviral diseases. Therefore, we aimed to identify prevalent bacterial and viral communities in *Aedes* mosquitoes from Pakistan.

**Methods:**

*Ae. aegypti* and *Ae. albopictus* were collected and from three different regions of Khyber Pakhtoonkhwa, Punjab and federal capital Islamabad. We isolated DNA and sequenced two pools of each species and conducted metagenomic analysis, identifying a variety of bacteria and viruses.

**Results:**

We found diverse bacterial and viral communities in both studied species. In *Ae. aegypti*, the most abundant bacterial species was *Klebsiella pneumoniae* followed by *Acinetobacter baylyi*. *Ae. albopictus* presented *Pseudomonas putida* as the most abundant bacterial species followed by *Brevundimonas diminuta*. Similarly in *Ae. aegypti*, we found that *Escherichia phage* HK639 was the most abundant viral species while in *Ae. albopictus*, it was *Lactobacillus phage* 2. It is important to mention that the prevalent viruses in both *Aedes* species belong to the *Siphoviridae* genus.

## 1 Introduction

Vector-borne diseases pose a significant burden on public health and economic growth of a country. Specifically, arboviral diseases are one of the main concerns due to the rapid increase in the incidence and vast geographical distribution. Chikungunya virus (CHIKV), Zika virus (ZIKV) and dengue virus (DENV) are some of the arboviruses of medical importance which need active surveillance and control^1^, ^2^, ^3^, ^4^. In humans, CHIKV, DENV, and ZIKV are transmitted through the bite of *Aedes aegypti* and *Aedes albopictus* mosquitoes^3^, ^4^, ^5^. In Pakistan, dengue virus has established endemicity, expanding to previously unaffected regions. In federal capital Islamabad, confirmed dengue cases increased from 2438 in 2023 to 3970 in 2024 with 15 deaths (Personal communication). This substantial increase in dengue cases, coupled with high mortality highlights a rapidly escalating trend of dengue fever severity within the region.

*Ae. aegypti* is widely distributed all over the world, particularly in tropical and subtropical urban areas^6^, ^7^. *Ae. albopictus* has successfully expanded its geographical range and adopted itself to subtropical or even colder temperate zones^3^, ^4^, ^8^.

Very few arboviral vaccines are currently qualified or licensed for human use, including those for yellow fever, Japanese encephalitis and dengue (Dengvaxia® [CYD-TDV]). The two main limitations in arboviral vaccines are low coverage and their safety, for example an increased risk of complicated dengue (shock and hemorrhage) in seronegative individuals. Other arboviruses, including Zika and chikungunya, do not have any approved vaccines as per now. Novel approaches target mosquito salivary proteins (or their components) or use other non-pathogenic insect-specific viruses to induce immunity through cross-protection. Nanoparticles and mRNA – based vaccines are also in pre-clinical or non-human trials stage^9^, ^10^. The only option that remains in absence of a vaccine or drug, is vector control. Despite vector control interventions, an increase in the geographical spread of *Aedes* species has been observed, mainly due to urbanization, globalization, pesticide resistance and climate change^11^, ^12^, ^13^.

Understanding the mosquito microbiome is crucial in controlling and inhibiting viral transmission. The microbiome associated with mosquitoes plays a crucial role in their susceptibility to infection, their ability to become infectious, the replication of viruses in their gut, their immune response, and various ecological factors^14^, ^15^. Therefore, a whole microbiome analysis of mosquito microbiome can give insights about the vector’s capability in disease transmission and proposing effective and safe biological control strategies^16^, ^17^. Biological control strategies have already been used in vector-borne diseases including the use of *Beauveria bassiana* (entomopathogenic fungi), mostly found on the surface of water where mosquitoes breed kills both larvae and adults of many mosquito species^18^, ^19^. Introduction of some strains of alpha-proteobacteria *Wolbachia* into its non-natural host *Ae. aegypti* impacts its competence by causing cytoplasmic incompatibility and shortening its life span^20^, ^21^, ^22^. Identifying and disrupting the symbiotic relationships between mosquitoes and their natural symbionts, or genetically modifying mosquitoes to control pathogen’s replication and transmission can be used as a safe biological control approach^23^, ^24^, ^25^.

The current metagenomic study was conducted to explore the microbiome of *Ae. aegypti* and *Ae. albopictus* by taking advantage of advanced high throughput molecular techniques. Metagenomic sequencing of the *Aedes* microbiome will be performed to assess the diversity and relative abundance of its microbial community. Current research can be helpful for designing effective control of *Aedes* mosquitos and *Aedes* borne diseases based on their microbiome.

## 2 Materials and Methods

### 2.1 Sample collection and preparation

**Fig. 1:**
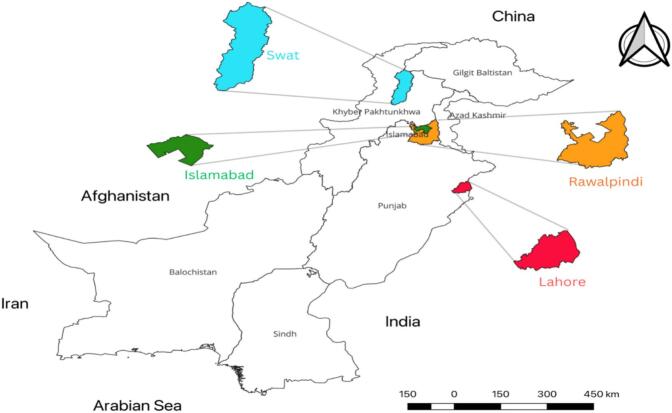
Sampling locations: Samples were collected from four regions, encompassing three geographical regions of Pakistan. The map is generated with the help of free and open-source software, qGIS (version 3.28). General Public License (GPL) developed by the Open-Source Geospatial Foundation Project (qgis.com).

**Fig. 2:**
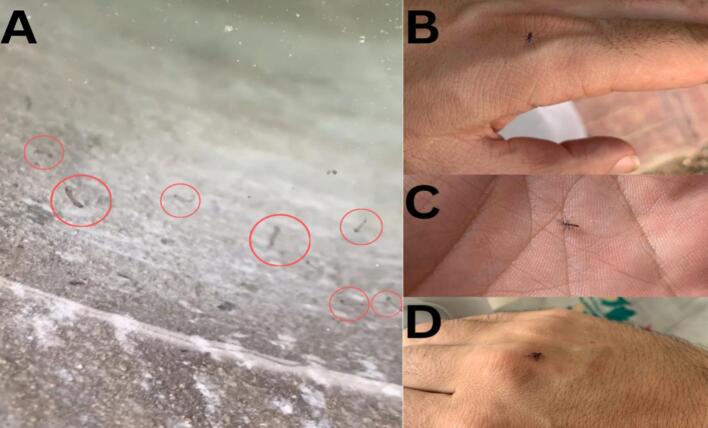
Sampling immature and adult life stages of Aedes mosquitoes. A. Larvae collection from a water container. B. Ae. albopictus female trying to bite one of co-author during collection. C. Ae. aegypti male captured by our collaborator. D. Ae. aegypti female smashed in due course of collection.

We obtained ethical approval of the study from the ethical committee of Department of Biosciences, COMSATS University Islamabad. This approval included the use of human blood for mosquito rearing, and human baiting to capture adult *Aedes* mosquitoes. We selected three dengue endemic regions of Pakistan for sample collection. We collected *Ae. aegypti* and *Ae. albopictus* from federal capital Islamabad, two districts of Punjab province (Rawalpindi and Lahore), and Swat district of Khyber Pakhtunkhwa from April-November 2022 (Fig. 1). We mainly collected immature life stages of these mosquitoes, however, when possible, we captured adults as well Fig. 2.

Mosquitoes were maintained inside an incubator at the department of Biosciences, maintaining optimum temperature (28–30 °C) and relative humidity (70–80 %). Adult mosquitoes were provided with a 10 % sugar solution (*ad libitum*). We followed the WHO protocol to rear adults and larvae with minute modifications as our previous studies mention them^26^.

Morphological identification of adults was carried out by naked eye using morphological key defined by Rueda 2004^27^. Female *Aedes* were separately pooled as 50 specimens/microtube and labelled properly. Samples were washed and cleaned with 70 % alcohol and treated with phosphate-buffered saline (PBS) and stored at −80 °C until nucleic acid extraction. Pools contained equal number of samples from all four locations.

### 2.2 DNA extraction

The mosquitos were mechanically homogenized with a pestle grinder within microtubes with nuclease free water and PBS for the preservation of nucleic acid. Samples were processed for DNA extraction using Qiagen® DNeasy Blood and Tissue kit (cat: 69504). The quantity of extracted DNA was measured using Qubit® 4 fluorometer (Invitrogen, Thermo Fisher, Scientific) to ensure its quality for the subsequent analysis. A negative control, without mosquito sample was run parallel to ensure the integrity of the whole extraction process.

### 2.3 Metagenomic sequencing and Bioinformatics analysis

DNA was then fragmented using suitable protocol and metagenomic library was prepared by attaching adapters to the DNA fragments to facilitate their binding and amplification. The library was then sequenced using high-throughput Illumina HiSeq instruments. After completion of sequencing, Illumina HiSeq/NovaSeq raw data was demultiplexed by index sequences, and paired end FASTQ files were generated for each sample. Adapter sequences and data with an average Phred quality score less than 20 were removed using Trimmomatic (v0.39) of the Knead data (v0.10.0) pipeline. Then, host-derived reads were filtered out by mapping them to the *Ae. aegypti* and *Ae. albopictus* reference genomes (GCF_002204515.2 and GCF_006496715.1, respectively) using Bowtie2 (v2.4.5). Flowchart details of library preparation is given in supplementary figure 1.

### 2.4 Assessment of sequencing depth and host depletion Efficacy

We filtered out host-derived reads by mapping the quality-controlled reads to the *Ae. aegypti* (RefSeq assembly accession: GCF_002204515.2) and *Ae. albopictus* (RefSeq assembly accession: GCF_006496715.1) reference genomes using Bowtie2 (v2.4.5) with default parameters. To quantify sequencing depth and the efficacy of this host depletion step, we report for each sample the raw read count, the number of reads passing adapter and quality trimming (Q-score > 30), and the number of reads that aligned to the respective host genome. The final non-host read count, which constitutes the input for all downstream microbiome analyses, is detailed in Table 1.Table 2..

**Table 1:** Sequencing data and pre-processing metrics.

Sample ID	Species	Raw reads (n)	QC Passed reads (n, %)	Reads mapped to host (n, %)	Non-host reads (n, %)
Sample_01	*Ae. albopictus*	42,816,318	40,235,501 (93.97 %)	38,102,726 (94.7 %)	2,132,775 (5.3 %)
Sample_02	*Ae. aegypti*	39,093,174	37,038,278 (94.74 %)	35,086,441 (94.7 %)	1,951,837 (5.3 %)

**Table 2:** Bacterial genera identified in *Ae. aegypti* and *Ae. albopictus*.

Percent (%) bacterial genera			
*Ae. aegypti*		*Ae. Albopictus*	
*Klebsiella*	52.630	*Pseudomonas*	88.152
*Acinetobacter*	34.740	*Brevundimonas*	11.266
*Enterobacter*	9.600	*Lampropedia*	0.582
*Stenotrophomonas*	3.021		
*Pantoea*	0.002		
*Elizabethkingia*	0.003		
*Staphylococcus*	0.004		

### 2.5 Taxonomic profiling of the microbiome

Taxonomic profiling was performed on the resulting non-host reads using MetaPhlAn4 (v4.0.0), which utilizes a library of clade-specific marker genes from ∼ 1 million microorganisms derived from NCBI reference genomes and species-level genome bins (SGBs). Briefly, the reads were mapped to this pan-microbial marker gene catalog using the integrated Bowti2 aligner. The relative abundance of each microbial species was calculated based on the average number of reads mapped to its unique marker genes. Any species for which less than 33 % of its marker genes were detected was considered absent and removed from the profile, as per the developer's recommendations.

### 2.6 Microbial diversity analysis

Microbial diversity (including richness, Shannon and Simpson values) was calculated using species abundance from the ‘calculate_diversity.R’ script of MetaPhlAn4. In addition, weighted and unweighted UniFrac distances, which are beta diversity values, were calculated based on the phylogenetic tree pre-calculated by the MetaPhlAn4 database. This whole process generated an extensive report of taxonomic classification of both bacteria and DNA viruses in *Ae. aegypti* and *Ae. albopictus* pools.

## 3 Results

DNA from two pools of *Aedes* mosquitoes (*Ae. aegypti* and *Ae. albopictus*) was successfully extracted. Concentrations from both samples were confirmed via Qubit® Fluorometer quantification, meeting the necessary criteria for metagenomic sequencing^28^. From these samples, a total of 15.6 gigabases of raw sequence data was generated. Following quality filtering and the removal of host-derived reads, a total of 12.1 Gb of reads were retained, which were then used for detailed microbiome analysis.

### 3.1 Relative microbial abundance in *Ae. Aegypti* and *Ae. Albopictus*

**Fig. 3:**
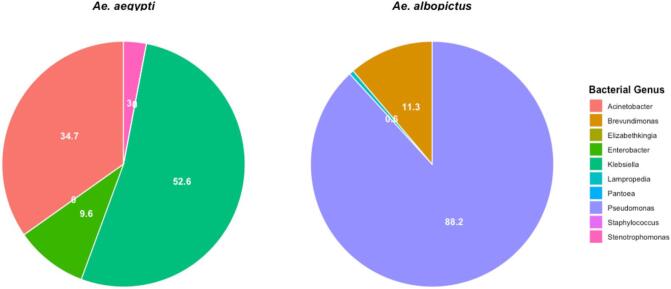
The relative abundance of bacterial genera in Ae. aegypti and Ae. albopictus. The most abundant are Acinetobacter and Pseudomonas in Ae. aegypti and Ae. albopictus respectively. Klebsiella and Enterobacter are in moderate abundance observed only in Ae. aegypti while other genera including Elizabethkingia, Pantoea, Staphylococcus and Stenotrophomonas are in low abundance.

Metagenomic analysis revealed the pattern of bacteria population in both pools (Fig. 3), table 2. Proteobacteria emerged as the most abundant phylum which displayed more than ∼ 99.99 % in *Ae. aegypti* to 100 % in *Ae. albopictus*. In comparison, Firmicutes and Bacteroidota were only found in *Ae. aegypti* with overall abundance less than 1 %.

Each species of mosquito sheltered unique genera of bacteria as; *Klebsiella*, *Stenotrophomonas*, *Acinetobacter*, *Staphylococcus*, *Elizabethkingia*, *Pantoea* and *Enterobacter* unique to *Ae. aegypti*. Similarly, *Pseudomonas*, *Brevundimonas*, and *Lampropedia* were unique to *Ae. albopictus*. Importantly, recently characterized bacterial specie *Lampropedia aestuarii* was identified for the first time in *Ae. albopictus* in the current study. Host-specific adherence of microbial population and diversity are likely to be due to the combination of host-specific factors, ecological interactions and environmental conditions^16^.

**Fig. 4:**
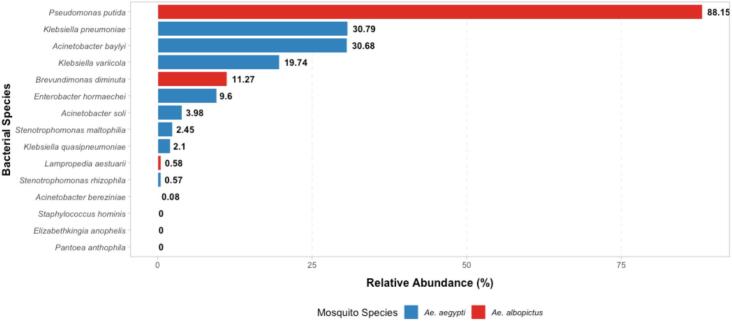
The relative abundance of bacterial species in Ae. aegypti and Ae. albopictus. The most abundant are Acinetobacter baylyi and Pseudomonas putida in Ae. aegypti and Ae. albopictus respectively. Klebsiella species and Enterobacter hormaechei are observed in moderate abundance only in Ae. aegypti Brevundimonas diminuta and Lampropedia aestuarii have moderate to low abundance in Ae. albopictus only.

The detailed analysis regarding the identified species within each mosquito revealed that in the *Ae. aegypti* sample, the genera *Acinetobacter* and *Klebsiella* were the most abundant. Specifically, the most common species were *Acinetobacter baylyi* (30.68 %), *Klebsiella pneumoniae* (30.79 %), and *Klebsiella variicola* (19.74 %), followed by *Acinetobacter soli* (3.98 %), *Klebsiella quasipneumoniae* (2.1 %), and *Acinetobacter bereziniae* (0.08 %) (Fig. 4**)**. In *Ae. albopictus* three species of genera *Pseudomonas*, *Brevundimonas* and *Lampropedia* were observed and *P. putida* emerged as the most abundant species (88.15 %) while *L. aestuarii* (1 %) was observed as the least abundant species as shown in Fig. 4.

### 3.2 Microbial Alpha diversity

Microbial alpha diversity measures species diversity in a specific ecosystem or sample. In the context of *Aedes,* microbial Alpha Diversity refers to the microbiomes associated with *Ae. aegypti* and *Ae. albopictus*. Microbial Alpha Diversity was measured by the Shannon Diversity Index, Observed Species Index, and Simpson Index (λ). Table 3 shows microbial diversity in the sample pool of *Ae. aegypti* and *Ae. albopictus.* The Observed Species Index refers to the number of distinct species present within each sample. It disregards the relative abundance of each species. A sample with a high value for the Observed Species Index demonstrates increased stability, imparting enhanced resistance against disturbances^29^, ^30^. The Simpson Index (λ) measures dominance, where a value of 1 indicates complete dominance by a single species (low diversity) and values approaching 0 indicate higher diversity.Table 4..

**Table 3:** Bacterial Alpha Diversity.

Sample pools	Observed species Index	Shannon Diversity index (H)	Gini Simpson Index
*Aedes aegypti*	12	1.4	0.73
*Aedes albopictus*	3	0.39	0.21

**Table 4:** Relative abundance (%) of prevalent virus species across *Ae. aegypti* and *Ae. albopictus* sample pools.

Genus	Species	*Ae. aegypti*	*Ae. albopictus*
*Siphoviridae*	*Escherichia phage* HK639	70.96	0
*Hk97virus*	*Enterobacterial phage* mEp390	22.56	0
*Myoviridae*	*Cronobacter phage*ENT47670	3.02	0
*Epsilon15virus*	*Salmonella virus* Epsilon15	1.2	0
*Siphoviridae*	*Enterobacteria phage* mEp237	1.16	0
*N15virus*	*Escherichia virus* N15	1.1	0
*Siphoviridae*	*Lactobacillus phage* A2	0	62.95
*Retroviridae*	*Human endogenous retrovirus* K	0	37.05

In our results, *Ae. aegypti* exhibited greater microbial diversity than *Ae. albopictus*, as evidenced by a higher Observed Species Index and Shannon Diversity Index, and a lower Simpson Index (λ).

In contrast, lower value of Observed Specie Index in *Ae. albopictus*, comprising three (03) species, suggests a microbial population that is relatively less diverse. Similarly, Shannon Diversity Index (H) considers both species diversity and evenness within an ecosystem or sample^31^. Table 3 shows that *Ae. aegypti* sample pool has bigger value (1.4), compared to *Ae. albopictus* sample pool further highlight the diversity and evenness in *Ae. aegypti*. Simpson’s index refers to the measurement of probability that two individuals selected randomly from a sample are related to the same species^32^. The lower value of Simpson Index shows higher microbial diversity. Higher value of Simpson Index (0.73) within *Ae. aegypti* signify lesser microbial diversity relative to *Ae. albopictus*^33^*.*

### 3.3 Relative abundance of DNA viruses?

**Fig. 5:**
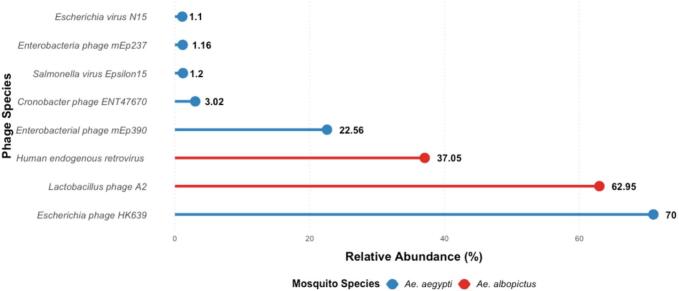
Relative abundance and percentage of DNA viral phages in Ae. aegypti and Ae. albopictus, with the most abundant phage Escherichia_phage_HK639(70.96%) and Lactobacillus_Phage_A2(62.95%) in both Ae. aegypti and Ae. albopictus respectively.

The presence of viruses in *Aedes* pose greater concern both for ecosystem and public health. The relative abundance (Fig. 5, table 4) of virus communities shows the presence and distribution of different DNA viruses within both sample pools. Genus *Siphoviridae* is predominant genus with 69.96 % abundance in *Ae. aegypti* and 62.95 % abundance in *Ae. albopictus*, *Hk97, Myoviridae, Epsilon15, Siphoviridae* and *N15* are the genera unique to *Ae. aegypti* while *Retroviridae* is the genus unique to *Ae. albopictus*. Furthermore, relatively significant abundant species *Human endogenous retrovirus K* belonging to the genus *Retroviridae* has been observed for the first time in *Ae. albopictus*. This is another novel finding of the current study which can make ground to explore vector competence of *Ae. albopictus*.

## 4 Discussion

Understanding microbiota of an organism which transmits parasites and their mutual interactions makes is extremely important. Therefore, we aimed to identify and compare the microbiota of two arbovirus vectors, *Ae. aegypti* and *Ae. albopictus* in Pakistan and its role in vector’s biology and competence for pathogen replication and transmission.

Metagenomic sequencing is a rapid and highly sensitive technique which can detect whole microbiomes in a pool of mixed community without any prior knowledge so, the genetic materials of all organisms, including bacteria, viruses, fungi, and archaea are isolated and screened in a single step^28^, ^34^. It is crucial to identify associated microbiota of mosquito species and its possible role in designing effective and safe biological vector control strategies.

We identified that Proteobacteria was the most abundant phylum found in both samples of *Ae. aegypti* and *Ae. albopictus* which align with previous studies^35^. Phylum Firmicutes and Bacteroidetes existed in low abundance and were unique only in *Ae. aegypti*^35^, ^36^, ^37^. But in our study Firmicutes and Bacteroidetes were not found in *Ae. albopictus.* It can be assumed that protobacteria is conserved microbiota of both *Aedes* species.

In Proteobacteria, two genera, *Pseudomonas* and *Klebsiella*, were abundant in our samples. *Pseudomonas* stimulates the immune system and produces antimicrobial peptides which might help them fight against viruses^38^. *Klebsiella* produce hemolysin, facilitate the digestion of food after absorption of blood^39^, ^40^. The most abundant bacterial species was *Klebsiella pneumoniae and Pseudomonas putida in Ae. aegypti* and *Ae. albopictus* respectively*. Klebsiella* species has been identified as an efficient symbionts and screened in all stages of life cycle of *Aedes* mosquitoes, playing an important role in larval growth and development^41^*.* Mosquera et al has also indicated that these bacterial species are transmitted by female mosquito while oviposition and larvae eventually acquire them after hatching. This survival and supportive behavior of vector might indicate significant role of *Klebsiella* species. Paul et al identified an enzyme, chitinase in one of the newly identified strain of *Pseudomonas putida* with effective role as biopesticide. Chitin is part of integument in larval as well as in adults of mosquitos^42^, ^43^*.* In this study, the presence of *Pseudomonas putida* as part of the *Aedes* microbiome should be further investigated to determine its role in larvicidal activities. A total of fifteen bacterial species were isolated from both samples; most were gram-negative, with the exception of one, *Staphylococcus hominis*. In contrast to previous studies, this gram-positive bacteria was found in lab raised *Ae. aegypti*^44^. *Lampropedia aestuarii* was observed for the first time in *Ae albopictus* in our study.

Among viruses, *Escherichia phage* HK639 was dominantly found *Ae. aegypti* and *Lactobacillus phage in Ae. albopictus.* Another notable finding of this study was the presence of *Human endogenous retrovirus K in Ae. albopictus.* The current study identified the *Siphoviridae* as the predominant family in the DNA virome in both mosquitoes which aligns with the previous studies, demonstrating that phages belonging to *Siphoviridae* are abundant in mosquitoes^45^, ^46^. However, the current study observed that both pools have variable viral communities, unique to each of the *Ae. aegypti* and *Ae. albopictus* mosquito. We identified Human endogenous retrovirus type-K (HERV-K) for the first time in *Ae. albopictus* mosquito. Currently this group of viruses is of great interest for researchers due its association with other human diseases. HERV detected in mosquito samples might be of human origin as the wild type mosquitoes feed on human blood, however, further investigations are needed to confirm if HERV exists naturally as part of the mosquitoes’ genome. Moreover, HERV was detected only in *Ae. albopictus* but not in *Ae. aegypti*. HERVs comprising 8 % of the human genome, are the remnants of the ancient germline with integrated retroviruses^47^, ^48^. Although HERVs are poorly explored, there are evidence defining their pathological roles in various diseases including autoimmune, cancer, and neurodegenerative diseases. HERV-K expression had been shown to be associated with the progression and mortality of SARS-CoV-2^47^, ^49^, ^50^. So, the presence of HERVs in *Ae. albopictus* might have a contributing role in mosquito competency and can be marker in disease severity.

Fifteen different species of bacteria were isolated from both *Aedes* species*.* Species related variations in microbial populations are attributed to the period and diet of the host mosquito^51^, ^52^. The microbiome of different mosquitoes species including *Ae. albopictus* and *Ae. aegypti* also affect their competence, and antagonism to some arboviruses^53^, ^54^, ^55^. The presence of diverse microbial compositions within *Ae. aegypti* mosquito population has significant implications on the rate of infection in different ecological and geographical contexts. Here in this study the Shannon index indicating unique microbial diversity of both mosquito species although both were collected from same geographical locations.

In this study, certain strains found in each sample exhibit distinct properties, showing that localized interactions might have a role in the susceptibility of mosquitoes to different pathogens. However, species that generally exist serve as primary indicators of the crucial microbiome characteristics that have a universal impact on the interaction between mosquitoes and pathogens. The prevalence rate of these species influences the microbial dynamics at a locality, impacting infection vulnerability. The observed fluctuations in bacterial and viral composition and prevalence indicate that each sample pool corresponds to different ecological behavior as both were sharing the common habitat. The observed variation in the rate of infectivity may be attributed to variation in ecological niche thereby provides an additional insight into the observed gap. Nonetheless, this information alone is not enough to find out their roles, interactions, or significant implications in the ecosystem. Further studies required to find out the impact of microbiota of these two particular vector species and its role in efficient replication and transmission of pathogens. But a comprehensive understanding of the subject matter can be used to improve the potency of a particular disease control program.

These observations show that ecological interactions or environmental factors might favor the growth and proliferation of specific microbial populations. The existence of the diverse microbial population within *Ae. aegypti* will have significant implications for apprehending the microbiome^56^, ^57^. Variation in microbial population may have an impact on the varying rates of infection in different geographical areas.

The microbiota of *Aedes* mosquitoes impact its fitness, competence and biological competition^58^. The microbiome of habitat and genetic diversities of mosquitoes impact how well and adult mosquito can be infected of virus and can potentially transmit it^59^. Invading pathogen has to interact with gut microbiome of adult mosquito. Therefore, impacting its replication. Few species of bacterial genera *Pseudomonas* has shown in *in vitro* studies that they have potential to inhibit viral replication by making antiviral metabolites in *Ae. albopictus*^60^. In our study we have identified genus *Pseudomonas* in *Ae. albopictus* which need further research to find its role as an antiviral agent.

Few studies have shown that microbiota of *Ae. aegypti* has a significant role for its susceptibility and transmission to DENV-2^61^. Similarly, *Ae. albopictus* is rapidly spreading in all continents and is a competent vector for dengue virus. Most of the studies are focusing on interaction of mosquitoes naturally occurring symbiont *Wolbachia* and its immunity but the impact of whole microbiome on its immune response and driving vector’s competence are less explore^62^, ^63^. Many studies are focusing on exploring the potential role of microbiome in insecticide resistance as this phenomenon is not only vertically transmitted but also maintained at gene level (Sahar Fazal et al, 2023, role of mosquito microbiome in Insecticide Resistance).

## 5 Conclusion

*Ae. aegypti* and *Ae. albopictus* are spreading to new geographical areas and thus becoming a substantial risk for human health. Their microbiota plays a critical role in shaping their immune system, competence and severity of disease transmission. Variation in microbiota is corelated with variation in competence of both species. The current study can help to suggest new microbial agents for the control and elimination of dengue vectors. These findings will help to design more effective and environmentally friendly vector control agents.

## 6 Data Availability

The raw sequencing data generated during this study are not publicly available due to restrictions in the original data sharing agreement associated with the sequencing service.. However, the processed data essential for reproducing all analyses and conclusions are provided within the main text or Supplementary Information of this article. This includes the relative abundance profiles of all detected microbial species and the detailed sequencing metrics and read counts for each sample.

## CRediT authorship contribution statement

**Shahzadi Asia Nadeem:** Writing – review & editing, Resources, Methodology, Investigation, Formal analysis. **Ijaz Ali:** Supervision, Methodology, Conceptualization. **Hazrat Hussain:** Writing – original draft, Methodology. **Ihsan Ullah:** Supervision, Methodology. **Wajid Ali:** Validation, Conceptualization. **Khalid J. Alzahrani:** Funding acquisition. **Hamid Ali:** Resources. **Zarak Imtiaz Khan:** Software, Methodology. **Kasim Sakran Abass:** Funding acquisition. **Rafi ur Rahman:** Writing – original draft, Supervision, Software, Methodology, Formal analysis, Conceptualization.

## Declaration of Competing Interest

The authors declare the following financial interests/personal relationships which may be considered as potential competing interests: Ijaz Ali reports administrative support and writing assistance were provided by COMSATS University Islamabad. If there are other authors, they declare that they have no known competing financial interests or personal relationships that could have appeared to influence the work reported in this paper.

## References

[1] Guzman M.G., Harris E. (2015). Dengue. The Lancet.

[2] Jones K.E., Patel N.G., Levy M.A. (2008). Global trends in emerging infectious diseases. Nature.

[3] Kraemer M.U.G., Sinka M.E., Duda K.A. (2015). The Global Distribution of the Arbovirus Vectors Aedes Aegypti and Ae..

[4] Kraemer M.U.G., Sinka M.E., Duda K.A. (2015). The Global Distribution of the Arbovirus Vectors Aedes Aegypti and Ae..

[5] Leta S., Beyene T.J., De Clercq E.M., Amenu K., Kraemer M.U.G., Revie C.W. (2018). Global risk mapping for major diseases transmitted by Aedes aegypti and Aedes albopictus. International Journal of Infectious Diseases : IJID : Official Publication of the International Society for Infectious Diseases.

[6] Espinal M.A., Andrus J.K., Jauregui B. (2019). Emerging and Reemerging Aedes-Transmitted Arbovirus Infections in the Region of the Americas: Implications for Health Policy. American Journal of Public Health.

[7] Souza-Neto J.A., Powell J.R., Bonizzoni M. (2019). Aedes aegypti vector competence studies: a review. Infection, Genetics and Evolution : Journal of Molecular Epidemiology and Evolutionary Genetics in Infectious Diseases.

[8] Paupy C., Delatte H., Bagny L., Corbel V., Fontenille D. (2009). Aedes albopictus, an arbovirus vector: from the darkness to the light. Microbes and Infection.

[9] Dimopoulos G. (2019). Combining Sterile and Incompatible Insect Techniques for Aedes albopictus suppression. Trends in Parasitology.

[10] Zheng X., Zhang D., Li Y. (2019). Incompatible and sterile insect techniques combined eliminate mosquitoes. Nature.

[11] Brady O.J., Golding N., Pigott D.M. (2014). Global temperature constraints on Aedes aegypti and Ae. albopictus persistence and competence for dengue virus transmission. Parasites & Vectors.

[12] Kraemer M.U.G., Reiner R.C., Brady O.J. (2019). Past and future spread of the arbovirus vectors Aedes aegypti and Aedes albopictus. Nature Microbiology.

[13] Ryan S.J., Carlson C.J., Mordecai E.A., Johnson L.R. (2019). Global expansion and redistribution of Aedes-borne virus transmission risk with climate change. PLOS Neglected Tropical Diseases.

[14] Gómez M., Martinez D., Muñoz M., Ramírez J.D. (2022). Aedes aegypti and Ae. albopictus microbiome/virome: new strategies for controlling arboviral transmission?. Parasites and Vectors.

[15] Minard G., Mavingui P., Moro C.V. (2013). Diversity and function of bacterial microbiota in the mosquito holobiont. Parasites & Vectors.

[16] Bernadus J.B.B., Pelealu J., Kandou G.D., Pinaria A.G., Mamahit J.M.E., Tallei T.E. (2023). Metagenomic Insight into the Microbiome and Virome Associated with Aedes aegypti Mosquitoes in Manado (North Sulawesi, Indonesia). Infectious Disease Reports.

[17] Ferreira, Q. R., Lemos, F. F. B., Moura, M. N., Nascimento, J. O. de S., Novaes, A. F., Barcelos, I. S., Fernandes, L. A., Amaral, L. S. de B., Barreto, F. K., & Melo, F. F. de. (2023). Role of the Microbiome in Aedes spp. Vector Competence: What Do We Know? In Viruses (Vol. 15, Issue 3). MDPI. 10.3390/v15030779. 10.3390/v15030779 PMC10051417 36992487

[18] Farenhorst M., Mouatcho J.C., Kikankie C.K. (2009). Fungal infection counters insecticide resistance in african malaria mosquitoes. Proceedings of the National Academy of Sciences of the United States of America.

[19] Scholte E.-J., Takken W., Knols B.G.J. (2007). Infection of adult Aedes aegypti and Ae. albopictus mosquitoes with the entomopathogenic fungus Metarhizium anisopliae. Acta Tropica.

[20] Mohanty I., Rath A., Mahapatra N., Hazra R.K. (2016). Wolbachia: a biological control strategy against arboviral diseases. Journal of Vector Borne Diseases.

[21] O’Neill S.L. (2018).

[22] Saridaki A., Bourtzis K. (2010). Wolbachia: more than just a bug in insects genitals. Current Opinion in Microbiology.

[23] Coutinho-Abreu I.V., Zhu K.Y., Ramalho-Ortigao M. (2010). Transgenesis and paratransgenesis to control insect-borne diseases: current status and future challenges. Parasitology International.

[24] Kean J., Rainey S.M., McFarlane M. (2015). Fighting arbovirus transmission: natural and engineered control of vector competence in Aedes mosquitoes. Insects.

[25] Saraiva R.G., Fang J., Kang S., Angleró-Rodríguez Y.I., Dong Y., Dimopoulos G. (2018). Aminopeptidase secreted by Chromobacterium sp. Panama inhibits dengue virus infection by degrading the E protein. PLoS Neglected Tropical Diseases.

[26] Rahman R.U., Souza B., Uddin I. (2021). Insecticide resistance and undfigureerlying targets-site and metabolic mechanisms in *Aedes aegypti* and *Aedes albopictus* from Lahore. Pakistan. Sci Rep.

[27] Pictorial key for the identification of mosquitoes (Diptera: Culicidae) associated with dengue virus transmission.

[28] Pérez-Cobas A.E., Gomez-Valero L., Buchrieser C. (2020). Metagenomic approaches in microbial ecology: an update on whole-genome and marker gene sequencing analyses. Microbial Genomics.

[29] Fatimawali, Kepel, B. J., Gani, M. A., & Tallei, T. E. (2020). Comparison of Bacterial Community Structure and Diversity in Traditional Gold Mining Waste Disposal Site and Rice Field by Using a Metabarcoding Approach. International Journal of Microbiology, 2020, 1858732. 10.1155/2020/1858732. 10.1155/2020/1858732 PMC6973193 31998378

[30] Schwartz M.W., Brigham C.A., Hoeksema J.D., Lyons K.G., Mills M.H., van Mantgem P.J. (2000). Linking biodiversity to ecosystem function: implications for conservation ecology. Oecologia.

[31] Tallei T.E., Fatimawali, Pelealu J.J. (2019). The data on metagenomic profile of bacterial diversity changes in the different concentration of fermented romaine lettuce brine. Data in Brief.

[32] Niode N.J., Adji A., Rimbing J., Tulung M., Tallei T.E. (2021). Composition and diversity of bacteria from giant asian honeybee Apis Dorsata gut. Biodiversitas.

[33] Pielou E.C. (1966). The measurement of diversity in different types of biological collections. Journal of Theoretical Biology.

[34] Gu W., Deng X., Lee M. (2021). Rapid pathogen detection by metagenomic next-generation sequencing of infected body fluids. Nature Medicine.

[35] Lin D., Zheng X., Sanogo B., Ding T., Sun X., Wu Z. (2021). Bacterial composition of midgut and entire body of laboratory colonies of Aedes aegypti and Aedes albopictus from Southern China. Parasites & Vectors.

[36] Boissière A., Tchioffo M.T., Bachar D. (2012). Midgut microbiota of the malaria mosquito vector Anopheles gambiae and interactions with Plasmodium falciparum infection. PLoS Pathogens.

[37] Rani A., Sharma A., Rajagopal R., Adak T., Bhatnagar R.K. (2009). Bacterial diversity analysis of larvae and adult midgut microflora using culture-dependent and culture-independent methods in lab-reared and field-collected Anopheles stephensi-an asian malarial vector. BMC Microbiology.

[38] Purtschert-Montenegro G., Cárcamo-Oyarce G., Pinto-Carbó M., Agnoli K., Bailly A., Eberl L. (2022). Pseudomonas putida mediates bacterial killing, biofilm invasion and biocontrol with a type IVB secretion system. Nature Microbiology.

[39] Albesa I. (1989). Klebsiella pneumoniae haemolysin adsorption to red blood cells. The Journal of Applied Bacteriology.

[40] Gundogan N., Citak S., Yalcin E. (2011). Virulence properties of extended spectrum β-lactamase-producing Klebsiella species in meat samples. Journal of Food Protection.

[41] Mosquera K.D., Martinez Villegas L.E., Pidot S.J. (2021). Multi-Omic Analysis of Symbiotic Bacteria Associated with Aedes aegypti Breeding Sites. Frontiers in Microbiology.

[42] Paul, M. K., & Mathew, J. (2023). *Mosquito Larvicidal Activity of Chitinase of Pseudomonas putida Mb 12 against the Human Vector Aedes aegypti*. *17*(December 2022), 403–410. 10.22207/JPAM.17.1.31.

[43] Paul, M. K., & Mathew, J. (2023). *Mosquito Larvicidal Activity of Chitinase of Pseudomonas putida Mb 12 against the Human Vector Aedes aegypti*. *17*(December 2022), 403–410. 10.22207/JPAM.17.1.31.

[44] Tuanudom R., Yurayart N., Rodkhum C., Tiawsirisup S. (2021). Heliyon Diversity of midgut microbiota in laboratory-colonized and fi eld-collected Aedes albopictus (Diptera : Culicidae): a preliminary study. Heliyon.

[45] Thannesberger, J., Rascovan, N., Eisenmann, A., Klymiuk, I., Zittra, C., Fuehrer, H., Scantlebury-manning, T., Hilaire, M. G., Austin, S., Landis, R. C., & Steininger, C. (2020). *Highly Sensitive Virome Characterization of Aedes aegypti and Culex pipiens Complex from Central Europe and the Caribbean Reveals Potential for Interspecies Viral Transmission*. 1–16. 10.3390/pathogens9090686 PMC7559857 32839419

[46] Thannesberger, J., Rascovan, N., Eisenmann, A., Klymiuk, I., Zittra, C., Fuehrer, H., Scantlebury-manning, T., Hilaire, M. G., Austin, S., Landis, R. C., & Steininger, C. (2020). *Highly Sensitive Virome Characterization of Aedes aegypti and Culex pipiens Complex from Central Europe and the Caribbean Reveals Potential for Interspecies Viral Transmission*. 1–16. 10.3390/pathogens9090686 PMC7559857 32839419

[47] Baldwin E.T., Götte M., Tchesnokov E.P. (2022). Human endogenous retrovirus-K (HERV-K) reverse transcriptase (RT) structure and biochemistry reveals remarkable similarities to HIV-1 RT and opportunities for HERV-K-specific inhibition. Proceedings of the National Academy of Sciences of the United States of America.

[48] Xue B., Sechi L.A., Kelvin D.J. (2020). Human Endogenous Retrovirus K (HML-2) in Health and Disease. Frontiers in Microbiology.

[49] Petrone V., Fanelli M., Giudice M. (2023). Expression profile of HERVs and inflammatory mediators detected in nasal mucosa as a predictive biomarker of COVID-19 severity. Frontiers in Microbiology.

[50] Temerozo, J. R., Fintelman-Rodrigues, N., dos Santos, M. C., Hottz, E. D., Sacramento, C. Q., de Paula Dias da Silva, A., Mandacaru, S. C., dos Santos Moraes, E. C., Trugilho, M. R. O., Gesto, J. S. M., Ferreira, M. A., Saraiva, F. B., Palhinha, L., Martins-Gonçalves, R., Azevedo-Quintanilha, I. G., Abrantes, J. L., Righy, C., Kurtz, P., Jiang, H., … Souza, T. M. L. (2022). Human endogenous retrovirus K in the respiratory tract is associated with COVID-19 physiopathology. Microbiome, 10(1), 65. 10.1186/s40168-022-01260-9. 10.1186/s40168-022-01260-9 PMC9024070 35459226

[51] Vogel K.J., Valzania L., Coon K.L., Brown M.R., Strand M.R. (2017). Transcriptome Sequencing reveals Large-Scale changes in Axenic Aedes aegypti Larvae. PLoS Neglected Tropical Diseases.

[52] Wang Y., Gilbreath T.M., Kukutla P., Yan G., Xu J. (2011). Dynamic gut microbiome across life history of the malaria mosquito Anopheles gambiae in Kenya. PloS One.

[53] Bian G., Zhou G., Lu P., Xi Z. (2013). Replacing a native Wolbachia with a novel strain results in an increase in endosymbiont load and resistance to dengue virus in a mosquito vector. PLoS Neglected Tropical Diseases.

[54] Moreira L.A., Iturbe-Ormaetxe I., Jeffery J.A. (2009). A Wolbachia symbiont in Aedes aegypti limits infection with dengue, Chikungunya, and Plasmodium. Cell.

[55] Walker T., Johnson P.H., Moreira L.A. (2011). The wMel Wolbachia strain blocks dengue and invades caged Aedes aegypti populations. Nature.

[56] Gratz, N. G. (2004). *Critical review of the vector status of Aedes albopictus*. 215–227. 10.1111/j.0269-283X.2004.00513.x. 10.1111/j.0269-283X.2004.00513.x 15347388

[57] Gratz, N. G. (2004). *Critical review of the vector status of Aedes albopictus*. 215–227. 10.1111/j.0269-283X.2004.00513.x. 10.1111/j.0269-283X.2004.00513.x 15347388

[58] Bennett K.L., Gómez-Martínez C., Chin Y. (2019). Dynamics and diversity of bacteria associated with the disease vectors Aedes aegypti and Aedes albopictus. Scientific Reports.

[59] Accoti A., Multini L.C., Diouf B. (2023). The influence of the larval microbiome on susceptibility to Zika virus is mosquito genotype-dependent. PLoS Pathogens.

[60] Manuscript, A. (2015). NIH Public Access. 6–13. 10.1016/j.cois.2014.07.004.The.

[61] Chen T.Y., Bozic J., Mathias D., Smartt C.T. (2023). Immune ‑ related transcripts , microbiota and vector competence differ in dengue ‑ 2 virus ‑ infected geographically distinct Aedes aegypti population. Parasites & Vectors.

[62] Moro C.V., Garrido M., Veiga J., Garrigós M., Puente J.M. (2023). *The interplay between vector microbial community and pathogen transmission on the invasive asian tiger mosquito*. Aedes Albopictus : Current Knowledge and Future Directions. July..

[63] Moro C.V., Garrido M., Veiga J., Garrigós M., Puente J.M. (2023). *The interplay between vector microbial community and pathogen transmission on the invasive asian tiger mosquito*. Aedes Albopictus : Current Knowledge and Future Directions. July..

