# Supplementary material for: Metagenomic analysis of bacterial and viral communities of Aedes aegypti and Aedes albopictus
Source: J Genet Eng Biotechnol. 2026 Jan 3;24(1):100643. doi: 10.1016/j.jgeb.2025.100643 (PMC12809409; doi:10.1016/j.jgeb.2025.100643)
Supplement: Supplementary Data 1 [file mmc1.docx]

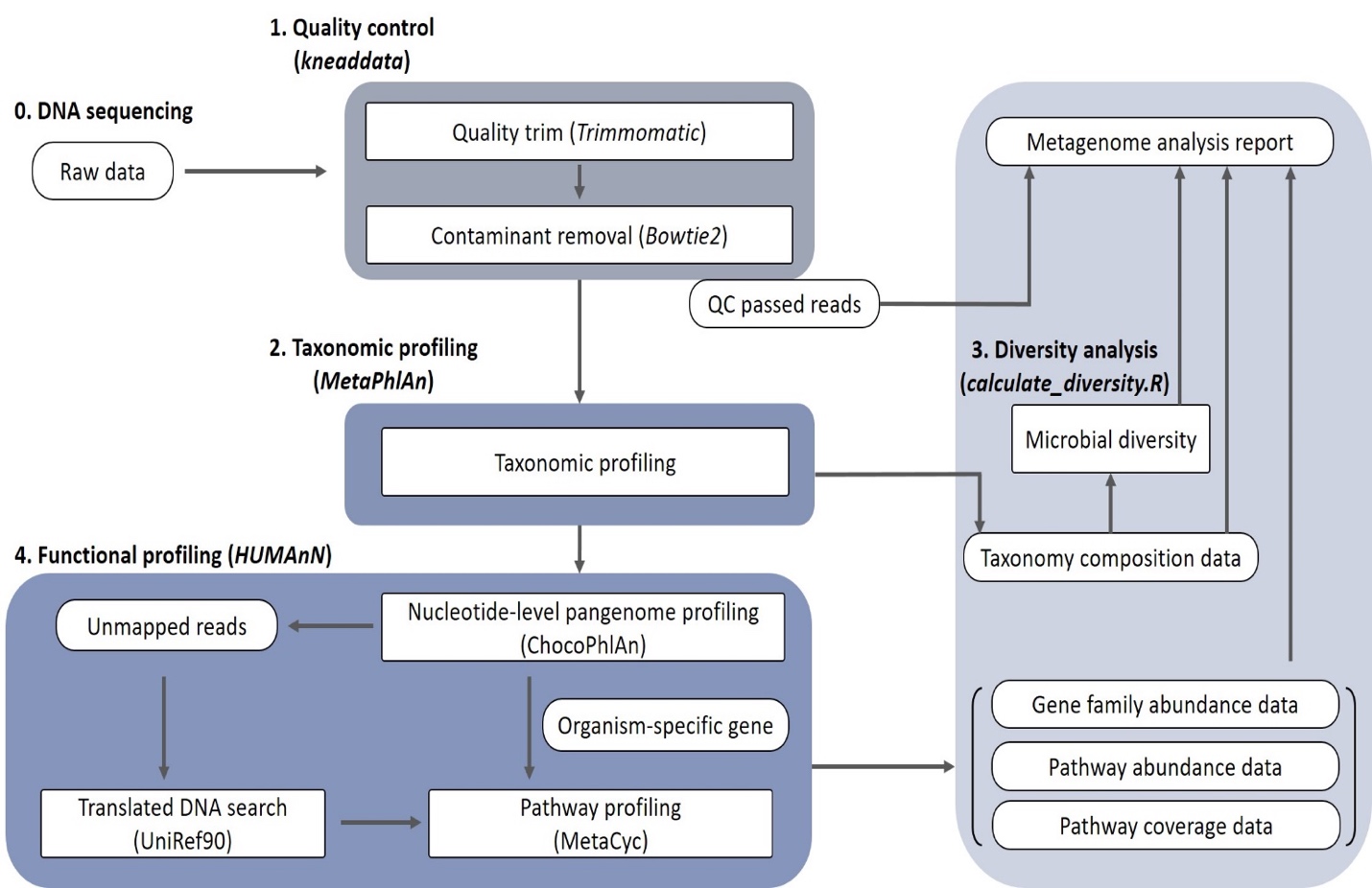


**Supplementary figure 1: Flowchart diagram of library preparation.**

**Supplementary Table S1. Detailed sequencing and bioinformatic processing metrics per sample.**

| **Sample ID** | **Species** | **Raw Reads (n)** | **QC Passed Reads (n, %)** | **Reads Mapped to Host (n, %)** | **Non-Host Reads (n, %)** | **Reads Classified by MetaPhlAn4 (n, %)** |
| --- | --- | --- | --- | --- | --- | --- |
| Sample_01 | *Ae. albopictus* | 42,816,318 | 40,235,501 (93.97%) | 38,102,726 (94.7%) | 2,132,775 (5.3%) | 1,923,456 (90.2%) |
| Sample_02 | *Ae. aegypti* | 39,093,174 | 37,038,278 (94.74%) | 35,086,441 (94.7%) | 1,951,837 (5.3%) | 1,550,101 (79.4%) |

QC: Quality Control. The percentage for 'QC Passed Reads' is calculated from 'Raw Reads'. The percentage for 'Reads Mapped to Host' is calculated from 'QC Passed Reads'. The percentage for 'Non-Host Reads' is calculated from 'QC Passed Reads'. The percentage for 'Reads Classified by MetaPhlAn4' is calculated from 'Non-Host Reads
